# African Swine Fever Circulation among Free-Ranging Pigs in Sardinia: Data from the Eradication Program

**DOI:** 10.3390/vaccines8030549

**Published:** 2020-09-21

**Authors:** Giulia Franzoni, Silvia Dei Giudici, Federica Loi, Daria Sanna, Matteo Floris, Mariangela Fiori, Maria Luisa Sanna, Paola Madrau, Fabio Scarpa, Susanna Zinellu, Monica Giammarioli, Stefano Cappai, Gian Mario De Mia, Alberto Laddomada, Sandro Rolesu, Annalisa Oggiano

**Affiliations:** 1 Department of Animal Health, Istituto Zooprofilattico Sperimentale della Sardegna, 07100 Sassari, Italy; silvia.deigiudici@izs-sardegna.it (S.D.G.); mariangela.fiori@izs-sardegna.it (M.F.); marialuisa.sanna@izs-sardegna.it (M.L.S.); paola.madrau@izs-sardegna.it (P.M.); susanna.zinellu@izs-sardegna.it (S.Z.); albertolad@LIVE.COM (A.L.); annalisa.oggiano@izs-sardegna.it (A.O.); 2 Epidemiological Veterinary Regional Observatory, Istituto Zooprofilattico Sperimentale della Sardegna, 09125 Cagliari, Italy; federica.loi@izs-sardegna.it (F.L.); stefano.cappai@izs-sardegna.it (S.C.); sandro.rolesu@izs-sardegna.it (S.R.); 3 Department of Biomedical Sciences, University of Sassari, 07100 Sassari, Italy; darsanna@uniss.it (D.S.); mfloris@uniss.it (M.F.); 4 Department of Veterinary Medicine, University of Sassari, 07100 Sassari, Italy; fscarpa@uniss.it; 5 National Swine Fever Laboratory, Istituto Zooprofilattico Sperimentale Dell’Umbria e Delle Marche, 06126 Perugia, Italy; m.giammarioli@izsum.it (M.G.); gm.demia@izsum.it (G.M.D.M.)

**Keywords:** African swine fever virus, eradication, free-ranging pigs, genotype I, haemadsorbing, next-generation sequencing, Sardinia

## Abstract

African swine fever virus (ASFV), the cause of a devastating disease affecting domestic and wild pigs, has been present in Sardinia since 1978. In the framework of the regional ASF eradication plan, 4484 illegal pigs were culled between December 2017 and February 2020. The highest disease prevalence was observed in the municipality with the highest free-ranging pig density, and culling actions drastically reduced ASFV circulation among these animals. ASFV-antibody were detected in 36.7% of tested animals, which were apparently healthy, thus, the circulation of low-virulence ASFV isolates was hypothesized. ASFV genome was detected in 53 out of 2726 tested animals, and virus isolation was achieved in two distinct culling actions. Two ASFV haemadsorbing strains were isolated from antibody-positive apparently healthy pigs: 55234/18 and 103917/18. Typing analysis revealed that both isolates belong to p72 genotype I, *B602L* subgroup X; phylogenetic analysis based on whole genome sequencing data showed that they were closely related to Sardinian ASFV strains collected since 2010, especially 22653/Ca/2014. Our data suggested the absence of immune-escaped ASFV variants circulating among free-ranging pigs, indicating that other elements contributed to virus circulation among these animals. Understanding factors behind disease persistence in endemic settings might contribute to developing effective countermeasures against this disease.

## 1. Introduction

African swine fever (ASF) is a devastating disease resulting in high mortality in domestic and wild pigs. It is currently present in Africa, Europe, Asia, and Oceania and causes dramatic losses in the pig sector due to its transcontinental spread and the lack of a licensed vaccine or treatment available [1,2,3]. The etiological agent is the ASF virus (ASFV), a large DNA virus belonging to the *Asfaviridae* family [4]. The ASFV genome size varies between 170 and 190 kbp length; differences are mainly due to copy numbers of five diverse multigene families (MGF), which seem to play a central role in the evasion of host defences [5]. ASFV field strains are segregated into 24 genotypes based on analysis of the variable region of the B646L gene, which encodes the major protein p72 [4,6]. 

ASFV was first introduced in Sardinia in 1978 and, despite several eradication programs, outbreaks in domestic pigs were detected until 2018, while the last PCR-positive sample in wild boar dates back to early 2019 [7,8]. All Sardinian isolates collected from 1978 to 2018 belong to genotype I ([9,10], ASF Virus Archive, Virology, Istituto Zooprofilattico Sperimentale (IZS) of Sardinia), whereas, in other areas of Europe, Asia, and Oceania, all the circulating ASFV isolates belong to genotype II [3,4], and all p72 genotypes are circulating in Africa [4]. Before this study, Sardinian ASFV strains were isolated only from domestic pigs presenting per-acute or acute clinical signs of the disease or from hunted/dead wild boar with unknown health status (ASF Virus Archive, Virology, Animal Health, IZS of Sardinia, Sassari, Italy). 

Natural reservoirs of the virus, such as naturally resilient wild Suidae (warthogs) or ticks of the genus *Ornithodorus,* are absent in Sardinia [11,12]. A central role played by wild boar in disease maintenance in Sardinia was never demonstrated [13,14], whereas several studies suggested a key role for free-ranging pigs in ASFV persistence in Sardinia [13,14,15], probably acting as a reservoir of the virus [8,16]. Thus, in the framework of the last ASF regional eradication plan (PE-ASF15-18; Regional Decree Number 5/6, 6 February 2015, and subsequent additions), several actions were conducted to eliminate illegal free-ranging pigs, coordinated by a special unit issued by the Sardinian government (“Unità di progetto”, “Project Unit”), as previously described [16]. High disease prevalence was detected during the first six months of these culling actions (December 2017–June 2018) [16], and veterinarians from the Animal Health Service (AHS) or specialized task force “GIV’ (“Gruppo di intervento veterinario”, “Veterinary special unit”) observed that these animals were apparently healthy and in good nutritional status at the time of culling. These pigs had probably recovered completely from infection and might have acted as carriers of ASFV, contributing to virus transmission and environmental contamination.

Similar scenarios have been described in Africa [17,18,19] and eastern Europe [20,21,22], and researchers suggested that low-virulence virus variants were circulating in those territories. In Latvia, an attenuated non-haemadsorbing (non-HAD) genotype II ASFV strain was isolated from a wild boar in 2017 [22]. A recent study described that an ASFV variant with reduced virulence, presenting a deletion of 14,560 base pairs at the 5′ end, was circulating among wild boar in North Estonia [23]. 

With this study, we aim to provide new information on the role of illegal free-ranging pigs in ASF persistence in Sardinia, with the hope of generating information useful to develop effective disease countermeasures. In the initial part of the work, a description of the geographic distribution and the ASFV prevalence in free-ranging pigs between 2017 and 2020 was provided. In the second part of the study, ASFV strains circulating among these illegal, apparently healthy, free-ranging pigs were characterized. We observed no evidence of non-HAD ASFV isolates in Sardinia, whereas two HAD strains were collected in two distinct culling actions: 103917/18 and 55234/18. These strains circulating among apparently healthy antibody-positive pigs were analysed through in vitro experiments on monocyte-derived macrophages (moMФ). Phylogenetic analyses and whole genome sequencing of 103917/18 and 55234/18 in a wider geographic context were also performed to evaluate their relationships with the other ASFV strains. 

## 2. Materials and Methods 

### 2.1. Ethics Statement

Healthy cross-bred pigs (*Sus scrofa domesticus*), 6–24 months old, were used for virus isolation from field samples, production/titration of virus stocks, and for in vitro experiments. Animals were housed at the Experimental Station of IZS of Sardinia (“Surigheddu”, Sassari, Italy). Animal husbandry and handling procedures were performed in accordance with the local ethics committee, in agreement with the Guidelines for the Use of Laboratory Animals issued by the Italian Ministry of Health. 

Samples from free-ranging pigs (*Sus scrofa*) were collected by veterinarians of the Italian AHS or the specialized task force GIV during the planned actions of the PE-ASF15-18 (and subsequent additions). Animals were culled after stunning, in agreement with the EU legislation on animal welfare (Council Regulation N°1099/2009). No free-ranging pigs were harmed or killed for this study; thus, approval of the ethics committee was not required.

### 2.2. Free-Ranging Pig Data

In the framework of the ASF eradication plan of Sardinia, 4484 free-ranging pigs were culled from December 2017 to February 2020, and almost 60% of them (older than three months of age) were randomly sampled. A total of 2491 sera were tested for ASFV antibody presence, whereas organs (mainly spleens) from 2726 free-ranging pigs were tested for virus presence.

The presence of ASFV antibodies was assessed using a commercial ELISA test (Ingezim PPA Compac^®^, Ingenasa, Madrid, Spain) as a screening test, confirmed by an immunoblotting test (IB), in accordance with the *Manual of Diagnostic Test and Vaccines for Terrestrial Animals*. IB^+^ samples were considered ASFV antibody-positive [24]_._ The presence of viral genome in organs was assessed by real time PCR, as previously described [25,26]. 

### 2.3. Malmquist Test

The presence of infectious ASFV was assessed in all PCR^+^ samples, using the Malmquist test, in accordance with the *Manual of Diagnostic Tests and Vaccines for Terrestrial Animals* [24]. In case of haemadsorption, presence of live ASFV was confirmed, and culture supernatant was collected and stored at −80 °C until propagation. In the absence of haemadsorption, the test was repeated by adding culture supernatants into fresh, two-day-old monocytes/macrophage monolayers. Only after three negative results, absence of live ASFV virus was confirmed [24]. 

### 2.4. Immunofluorescence Staining Test

All PCR^+^ Malmquist^−^ samples were screened for the presence of non-HAD ASFV isolates. Culture supernatants of Malmquist^−^ samples were inoculated into fresh, two-day-old monocytes/macrophage monolayers, using 48-well plates. After five days, immunofluorescence staining was performed, in accordance with the *Manual of Diagnostic Tests and Vaccines for Terrestrial Animals* [24], using an FITC-conjugated anti-ASFV polyclonal antibody (National Swine Fever Laboratory, Perugia, Italy), diluted 1:200 in PBS, as previously described [26].

### 2.5. Viruses 

Two strains were characterized in these studies: 103917/18 and 55234/18, isolated in 2018 from free-ranging pigs in two distinct culling actions. Details are provided in Table S1. These strains were compared in vitro to the virulent Sardinian field strains 26544/OG10 (isolated from naturally infected pigs during ASF outbreaks in Sardinia in 2010) (ASF Virus Archive, Virology, Animal Health, IZS of Sardinia, Sassari, Italy) and the attenuated NH/P68 (kindly provided by the EU ASF Reference Laboratory CISA-INIA, Madrid, Spain). ASFV strains were propagated in vitro by inoculation of sub-confluent monolayers of two-day-old monocytes/macrophage cultures, prepared as described before, using a 25 cm^2^ flask (Corning, Corning, NY, USA) [24,26]. After two or three days of incubation at 37 °C in 5% CO_2_, supernatants were collected and pooled with a freeze-thawed cell lysates. The resultant pool was clarified by centrifugation at 3000× *g* for 15 minutes (min), divided into aliquots, and stored at −80 °C. Mock-virus supernatants were prepared in an identical manner from uninfected cultures. Titres of HAD ASFV isolates (103917/18, 55234/18, 26544/OG10) were obtained by serial dilution of virus suspensions on two-days-old monocyte/macrophage cultures (using 96-well plates), followed by haemadsorption. Viral titres of NH/P68 were obtained by serial dilution of the virus suspension on monocytes/macrophages (using 96-well plates), followed by immunofluorescence staining, as described before. Viral titres were determined using the Spearman–Kärber formula [24,26].

### 2.6. Generation of Porcine Monocyte-Derived Macrophages 

Macrophage cultures were obtained from blood leukocytes using Petri dishes and media supplemented with 50 ng/mL of recombinant human M-CSF (hM-CSF) (Thermo Fisher Scientific, Waltham, MA, USA), as previously described [27]. MoMФ were re-suspended in RPMI-1640 supplemented with 10% foetal bovine serum (FBS), 100 U/mL penicillin, and 100 μg/mL streptomycin (complete RPMI, cRPMI) and seeded in 12-well plates (Greiner CELLSTAR, Sigma-Aldrich, Saint Louis, MO, USA) (7–8 × 10^5^ live cells per well) or 96-well plates (1 × 10^5^ live cells per well). After seeding, cells were incubated at 37 °C 5% CO_2_ for a further 24 h before infection. 

### 2.7. Growth Kinetics of ASFV in MoMФ 

MoMФ were cultured in 12-well plates. Cells were infected with 26544/OG10 or 103917/18 or 55234/18 ASFV at a multiplicity of infection (MOI) of 0.01. After 90 min incubation at 37 °C 5% CO_2_, virus inoculum was removed, the cells were washed with un-supplemented RPMI-1640 medium, and fresh cRPMI was added to the wells. Cells were cultured at 37 °C 5% CO_2_, and supernatants were collected at 0, 24, 48, and 72 h post-infection (hpi). Supernatants were clarified from cellular debris by centrifugation at 2000 × g for 3 min and then stored at −80 °C until assessment of infectious virus levels by titration, as described above. 

### 2.8. Impact of ASFV Infection on MHC I Expression and Viability of MoMФ 

MoMФ were cultured in 12-well plates (to assess ASFV impact on MHC I expression) or 96-well plates (to assess ASFV impact on moMФ viability). Culture medium was removed, and cells were infected with 26544/OG10, 103917/18, 55234/18, or NH/P68 ASFV at a multiplicity of infection (MOI) of 1. Mock-infected controls were included in every experiment. After 90 min incubation at 37 °C 5% CO_2_, virus inoculum was removed, cells were washed with un-supplemented RPMI-1640 medium, and fresh cRPMI was added to the wells [27]. 

The 21 hpi, ASFV impact on MHC I surface expression was assessed by flow cytometry, as previously described, with slight modifications [27]. 

MHC class I antibody (JM1ER; Bio-Rad Antibodies, Kidlington, UK) and RPE-conjugated goat anti-mouse IgG-Fc polyclonal antibody (Thermo Scientific Pierce) were used to assess MHC I surface expression, whereas anti-p72-FITC antibody (18BG3, Ingenasa) was used to evaluate intracellular levels of ASFV protein p72. Cells were analysed with a FACS Celesta (BD Biosciences, Franklin Lakes, NJ, USA), and 5000 live moMФ were acquired. Analysis of data was performed using BD FACS Diva Software (BD Biosciences) by gating on viable moMФ and then assessing the staining for MHC class I and ASFV late p72 proteins. Gates for p72 protein were set using the mock-infected controls, and then the mean fluorescence intensities (MFI) of PE (MHC class I) of un-infected (mock), bystander (p72^−^), and infected (p72^+^) moMФ were determined, as previously described [27].

ASFV effect on cell viability was assessed at 21 hpi, using a non-radioactive cytotoxicity assay. LDH (lactate dehydrogenase) levels in culture supernatants were quantified using a Cytotox 96^®^ Non-Radioactive Cytotoxicity Assay (Promega, Madison, WI, USA) according to the manufacturer’s instructions. A lysis solution provided by the manufacturer was used as a positive control, whereas mock-infected moMФ were used as a negative control. Absorbance was read at 492 nm using an Epoch microplate reader (BioTek, Winooski, VT, USA).

### 2.9. DNA Extraction, Quantification, and Amplification

Viral DNA was extracted from cell culture supernatant using a QIAmp UltraSens Virus Kit (Qiagen, Hilden, Germany), following the manufacturer’s instructions. DNA quantification was performed using an Epoch microplate spectrophotometer (BioTek) and a Qubit 3.0 Fluorometer (Thermo Fisher Scientific), according to the manufacturer’s instructions.

### 2.10. Genome Sequencing 

Viral libraries were generated according to manufacturer’s protocols, using Nextera DNA Flex Library Prep kit (Illumina Inc., San Diego, CA, USA) starting from a minimum DNA input of 50 ng. Using a Nextera DNA Flex library prep, quantifying and normalizing individual libraries generated in the same experiment were not necessary. To achieve optimal cluster density, equal library volumes were pooled, and pools were quantified before sequencing using a Qubit fluorescent dye method. To check library quality, 1 μL of pooled libraries was run on an Agilent 2100 Bioanalyzer with a High Sensitivity DNA kit. Library size profiles had an average fragment size of 600 bp. Sequencing was carried out on Novaseq 6000 (Illumina) diluting libraries to the starting concentration for the system. A median coverage of 250 was obtained (AMES Group, Centro Polidiagnostico Stumentale S.r.l, Napoli, Italy).

Genome data processing was performed using an in house bioinformatic pipeline. The bcl2fastq program [28] was used to convert BCL (binary base call) files generated by the sequencing systems to standard FASTQ file formats. Trim Galore [29] was used to quality trim the data and remove sequencing adaptors. The reads were then aligned to the pig reference genome (*Sus scrofa* 10.2 [30]) using the bwa-mem algorithm [31]. Aligned bam files were sorted and indexed with samtools [32] and deduplicated with Picard-tools [33]. To obtain high-quality variants, freebayes [34] was used to call variants for each sample, using the *KX354450.1* sequence as reference genome (parameters: “*--ploidy 1 -X -u -m 20 -q 20 -F 0.2*”).

### 2.11. Phylogenetic and Typing Analyses

Phylogenetic analyses were performed independently on two different datasets of sequences. The first included 48 ASFV whole genomes from Africa, Asia, and Europe; 46 of them were taken from GenBank, and two were obtained during the present study. Details are provided in Table S1. The second dataset was a subset of the first one. All the genomes (16) from Sardinia were included in the second dataset. In particular, the strains 103917/18 and 55234/18 isolated in the present study were compared to all Sardinian strains fully sequenced thus far available (see details in Supplementary Table S1). Genome alignment was carried out using the algorithm L-INS-I implemented in the software Mafft 7.427 [35]. After the alignment, a manual check of the dataset was performed using Unipro UGENE v.35 [36]. A Bayesian inference for phylogenetic trees was performed using the software MrBayes 3.2.7 [37] setting as model parameters: NST = 6, rates = invgamma, ngammacat = 4. Two independent runs, each consisting of four Metropolis-coupled Markov chain Monte Carlo (MCMC) chains (one cold and three heated chains), were performed simultaneously for 5,000,000 generations, sampling trees every 1000 generations. The first 25% of the 10,000 sampled trees was discarded as burn-in. Runs were carried out by means of the CIPRES Phylogenetic Portal [38]. In order to verify the convergence of chains, we checked that the average standard deviation of split frequencies (ASDSF) approached 0 [37], and the potential scale reduction factor (PSRF) was around 1 [39], following Scarpa et al. [40]. Phylogenetic trees were visualized using FigTree 1.4.0 [41]. Typing was performed analysing *B646L*, *B602L*, and *EP402R* genes.

The C-terminal end of the B646L gene of 103917/18 and 55234/18 isolates was aligned with the corresponding region of the international strains belonging to all 24 genotypes and retrieved from GenBank. Phylogeny was estimated in MEGA 7 [42] via the neighbour-joining model of nucleotide substitution. Statistical support for specific clades was obtained via 1000 bootstrap replicates. The amino acid sequence of the *B602L* and the *EP402R* genes was compared with the corresponding regions of all Sardinian fully sequenced strains (Table S1). Deletions in these two genomic regions were confirmed by Sanger sequencing using the primers and the methods described previously [9].

### 2.12. Data Analysis

For the purpose of this work, an ad hoc database was built to collect data about location, time (day, month, year), number of culled animals, animals tested for ASFV antibody and virus presence, and ASFV-positive free-ranging pigs. Epidemiological statistical analyses were carried out to calculate antibody-prevalence and virus-prevalence by year (2017–2020) and location (municipalities) of the culling actions, with associated 95% confidence intervals (CI) for a proportion based on the central limit theorem for binomial outcomes. The basic reproduction number (*R*_0_), formally defined as the average of secondary cases that would result from the introduction of one infectious in a total susceptible population [43], was calculated based on the time of the first culling actions in each municipality. Only those locations where the virus has been detected at least one time were considered in the *R*_0_ estimation. 

The *R*_0_ parametrization proposed by Becker (1989), based on susceptible population at the start (*S*_0_) and at the end (*S_f_*) of the epidemic, was applied [44]. Considering the total population size *N* and the total number of cases *C* (i.e., individuals that have been infected), *R*_0_ was calculated as follows:

(1) R0≅N−1C*ln{S0+12Sf−12}

Basically, if we assume that the proportion of individuals that tested negative for the infection (i.e., PCR-negative and antibody-negative) is equal to the proportion of susceptible population (*s*), *s* can be estimated as follows:

(2) $$s_{t}=\;\underset{t}{\sum }\frac{S_{t}}{N_{t}}\;$$

where *S_t_* is the number of susceptible animals among those tested at time *t* and *N_t_* is the number of animals that were tested. 

The standard errors of the estimated *R*_0_ parameters are as follow:

(3) $$SE\left(R_{0}\right)=\frac{N-1}{C}*\sqrt{\overset{S_{0}}{\underset{j=S_{f}+1}{\sum }}\frac{1}{j^{2}}+\frac{CR_{0}^{2}}{{\left(N-1\right)}^{2}}}$$

In vitro experiments were performed in technical duplicate and repeated with three (growth curves, MHC I expression) or four (cytotoxic test) different blood donor pigs. Baseline data distribution was evaluated based on Shapiro–Wilk test. Differences within normally distributed samples were analysed with the parametric student T-test with analysis of variance (ANOVA) followed by Bonferroni method for multiple comparisons. All statistical analyses were performed using *R-software*, version 3.6.2 (R-Foundation for Statistical Computing, Vienna, Austria), and a level of *p* < 0.05 was considered as statistically significant. GraphPad Prism 8.01 (GraphPad Software Inc., La Jolla, CA, USA) was used for graphical analyses and representations.

## 3. Results

### 3.1. Distribution of ASFV among Illegal Pigs in Sardinia: Data from Eradication Program

**Figure 1:**
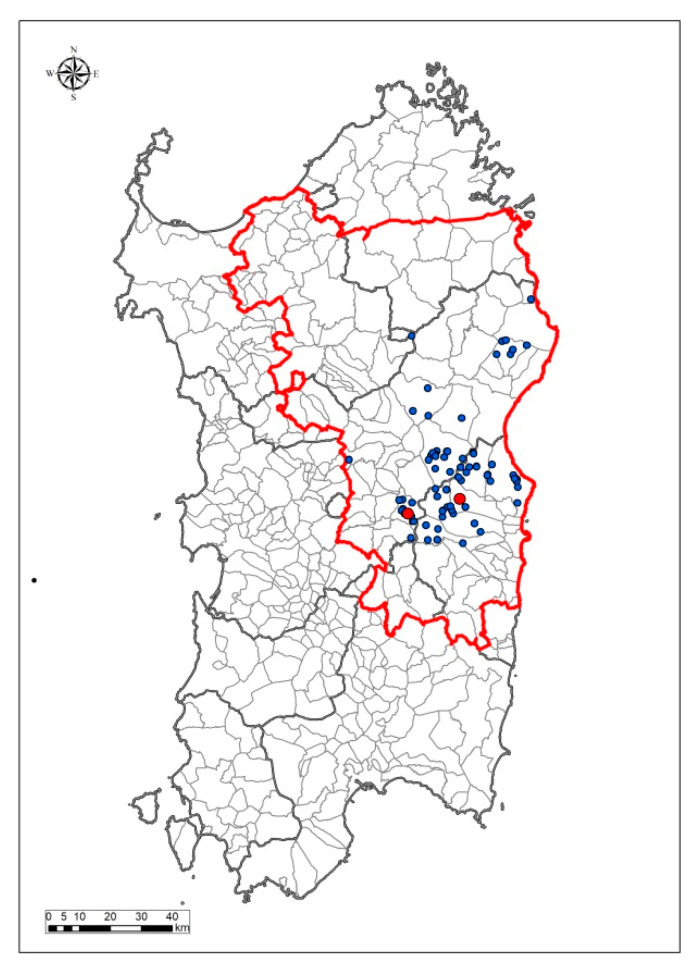
Map representing the Sardinian municipalities of the free-ranging pig culling actions (blue and red dots). The 55234/18 and the 103917/18 were isolated from samples collected in Desulo and Talana (red dots), both inside the wild boar infected zone (red line).

A total of 47 culling actions against free-ranging pigs were performed between 2017 and 2020 in 18 municipalities, all located in the middle of Sardinia (Figure 1). All these actions were carried out inside the “wild boar infected zone”, a 9000 km^2^ inner area of the island, where stronger actions against ASFV in sylvatic populations have been adopted since 2017 [14]. Laboratory analysis revealed a high prevalence of antibody-positive illegal free-ranging pigs, which decreased over the years: 61.9% (confidence intervals (CI) 95% = 56.6–66.9) in 2017, 40.4% (CI 95% = 37.9–42.9) in 2018, 14.8% (CI 95% = 11.7–18.3) in 2019, and 14.4% (CI 95% = 9.5–20.5) during the culling action of 2020, as reported in Table 1. ASFV genome was overall detected in fewer than 2% (CI 95% = 1.4–2.5) of the tested pigs (PCR-positive) with a virus prevalence of 3.8% (CI 95% = 2.1–6.3) during December 2017, 2.2% (CI 95% = 1.6–3.0) in 2018, 0.2% (CI 95% = 0.0–1.1) in 2019, and 0% in 2020 (CI 95% = 0–0) (Table 1). Despite the presence of ASFV antibodies, these animals had a good nutritional status and were apparently healthy at the time of culling, without clinical signs reported by veterinarians. Representative images are displayed in Figure S1.

The main communities of about 1400 and 700 illegal free-ranging pigs were found in Orgosolo and Urzulei municipalities, respectively. Medium-sized groups of animals (average of 200–350 animals) were located in seven other municipalities (Arzana, Baunei, Desulo, Irgoli, Nuoro, Talana, Villagrande Strisaili). All the other culling actions were performed on small groups of animals (<20 pigs). As displayed in Table 2, the highest antibody-prevalence was detected in those municipalities where the density of illegal free-ranging pigs (animals/km^2^) was highest (Arzana, Desulo, Orgosolo, Talana), as estimated by Bosch et al. [45]. PCR^+^ pigs were detected in five municipalities: Arzana, Desulo, Orgosolo, Talana, and Villagrande Strisaili (see Table 2). The basic reproduction number *R*_0_ was calculated only for those municipalities where the ASF was detected at least one time. 

The maximum parameter values were estimated in Desulo (*R*_0_ = 2.67, SE = 0.98) and Orgosolo (*R*_0_ = 2.23, SE = 0.95), while the lowest *R*_0_ was associated with Baunei municipality, where very low seroprevalence and no PCR^+^ samples were found. Comparing these estimations with those recently obtained by Bosch et al. about the number of contacts between animals [45] and thus the animal interaction index, the maximum values of *R*_0_ corresponded to those areas with maximum animal density and contact rates (Orgosolo and Desulo). The same municipalities have been previously defined as the main problematic areas for ASF persistence given the evidence of rural socio-economic conditions and the ASF endemic context [8,14,16].

Only 53 of 2726 illegal pigs had ASFV genome in their organs. As showed in Figure S2 and Table S2, PCR^+^ samples presented high Ct values (mean = 35.05, SD = 4.62), indicating the presence of low levels of ASFV genome. PCR^+^ samples were subsequently assayed for virus isolation with Malmquist test. Infectious ASF virions were detected in only 10 samples, collected in two distinct culling activities: Desulo (11/06/2018) and Talana (17/12/2018), as displayed in Figure 1 and Table S2. In total, 43 out of 53 PCR^+^ samples were Malmquist^−^; Ct values were very high (mean = 36.3, SD = 2.32) (Figure S2), suggesting that no live virus was present in these organs. Nevertheless, all the Malmquist^−^ samples were assayed with immunofluorescence staining in order to exclude the presence of non-HAD ASFV strains. All the immunofluorescence staining tests gave negative results, strongly suggesting the absence of non-HAD ASFV strains among free-ranging pigs in Sardinia.

### 3.2. Isolation and In Vitro Characterization of Two Isolates Collected from Asymptomatic Free-Ranging Pigs

Virus isolation was achieved in 10 PCR^+^ organs (nine spleens and one lung), and 70% of these organs belonged to seropositive pigs, as displayed in Table S2 and Figure S3. One isolate per culling action was selected, amplified, and used for further analyses: 55234/18(6) (Desulo) and 103917/18(4) (Talana). The 55234/18 isolated from a fattening pig presented a strong PCR result (Ct 23.58) and low antibody titre, whereas 103917/18 was collected from an adult pig and presented a weak PCR result (Ct 34.84) and high antibody titre (Table S2 and Figure S3). Both isolates were HAD, with no visible differences with the virulent Sardinian isolate 26544/OG10, as shown in Figure S4.

**Figure 2:**
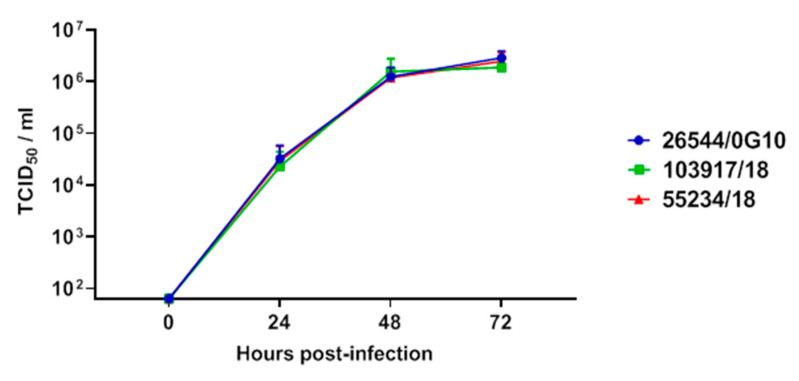
Growth kinetic in macrophages of the two ASFV strains collected from illegal free-ranging pigs. moMΦ were infected with the strains under study (103917/14 or 55234/18) or the virulent 26544/OG10 ASFV strains using a multiplicity of infection (MOI) of 0.01. At 0, 24, 48, and 72 h pi, duplicate samples were collected, and infectious viral progeny in culture supernatants were assessed by titration (TCID_50_/mL). The sensitivity of this assay was 1.8 log_10_ 50% haemadsorbing doses/mL (TCID_50_/mL). The mean values and the standard deviation from three independent experiments utilizing different animals are shown. At each time-point, values of 103917/14 and 55234/18 and 26544/OG10 viral titres were compared using the analysis of variance (ANOVA) for repeated measures.

In vitro assays were performed to compare the two isolates in this study with the Sardinian virulent strain 26544/OG10. The latter presented remarkable virulence in vivo; intramuscular inoculation with only 10 TCID_50_ of 26544/OG10 led to death in domestic pigs after 10–14 days (Gian Mario De Mia, unpublished data). A kinetic analysis of the infection with 103917/18, 55234/18, and 26544/OG10 ASFV strains was performed in moMФ using a MOI of 0.01. Viral titres in culture supernatants were measured longitudinally, at 0, 24, 48, and 72 hpi. All the tested Sardinian isolates replicated efficiently and without differences in moMФ (Figure 2), with a growth kinetic similar to what we previously observed for 22653/Ca/2014 (22653/14) [27].

**Figure 3:**
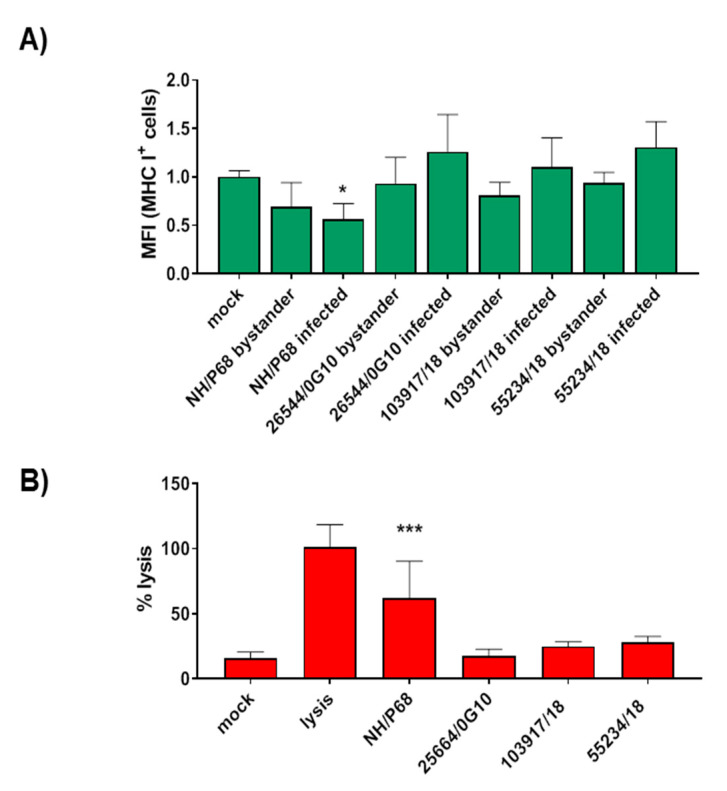
Effect of 103917/14 and 55234/18 ASFV infection on MHC class I expression and viability of macrophages. moMΦ were infected with the low-virulence NH/P68, the virulent 26544/OG10, or the isolates under study 103917/18 and 55234/18 using an MOI of 1, alongside mock-infected controls. At 21 hpi, surface expression of MHC class I and moMФ viability were assessed. (**A**) MHC class I expression (MFI) and intracellular levels of ASFV p72 were assessed by flow cytometry; MFI data are presented as fold change relative to the mock-infected condition. Values of ASFV-infected or bystander macrophages were compared to the corresponding mock-infected control. (**B**) moMФ viability was assessed using a non-radioactive cytotoxic assay, which quantifies lactate dehydrogenase (LDH) levels in culture supernatants. For both panels, mean values and standard deviation from three (**A**) or four (**B**) independent experiments utilizing different animals are shown as green and red bars with whiskers lines, respectively. Analysis of variance and relative Bonferroni method for multiple comparisons were applied to evaluate difference between samples; *** *p* < 0.001, * *p* < 0.05.

To further investigate potential differences between these strains, we investigated the effects of infection on MHC class I expression and moMФ viability. Our previous study demonstrated that infection with attenuated genotype I NH/P68, but not the virulent Sardinian strains 22653/14, induced MHC I down-regulation in infected macrophages [27]. moMФ were infected with 55234/18, 103917/18, the virulent 26544/OG10, or the attenuated non-HAD NH/P68, using an MOI = 1, alongside mock-infected controls. At 21 h pi, the surface expression of MHC I and the intracellular levels of late viral protein p72 were assessed by flow cytometry, and, as expected, infection with NH/P68 resulted in MHC I down-regulation in infected cells [27]. On the contrary, as displayed in Figure 3A, the two strains isolated from free-ranging pigs (103917/18 and 55234/18) and the virulent 26544/OG10 had a similar impact on MHC I expression; infected cells (p72^+^) presented a slightly higher, but not statistically significant, MHC class I expression (mean fluorescence intensity, MFI) compared to bystander (p72^−^) and mock-infected cells (Figure 3A), similar to what we previously observed for 22653/14 [27]. We then assessed the impact of ASFV infection on moMФ viability. At 21 hpi, cells viability was determined using a non-radioactive cytotoxic test. Lactate dehydrogenase (LDH) levels in culture supernatants of 26544/OG10, 103917/18, and 55234/18 –infected moMФ were not statistically significantly different to those of the mock-infected control. On the contrary, NH/P68-infected moMФ presented higher levels of LDH in culture supernatants, indicating that NH/P68, but not the two strains under study, has a stronger impact on moMФ viability (Figure 3B). Overall, these in vitro studies did not reveal differences between the two “free-ranging pig” isolates and virulent Sardinian strains.

### 3.3. Genetic Characterization of Two Isolates Collected from Asymptomatic Free-Ranging Pigs

The analysis of the C-terminal end of the B646L gene (coding for p72) revealed that both 103917/18 and 55234/18 belonged to genotype I, similar to all other Sardinian ASFV isolates collected since 1978 (ASF Virus Archive, Virology, IZS of Sardinia, Sassari, Italy) (data not shown). Analysis of the *B602L* and the *EP402R* genes showed that both 103917/14 and 55234/18 clustered in subgroup X of the *B602L* gene and presented a six amino acid repeat deletion at the C-terminal of the CD2v protein (encoded by *EP402R* gene), as with almost all the Sardinian isolates collected since 1990 (“modern” Sardinian isolates) (Figures S5 and S6).

**Figure 4:**
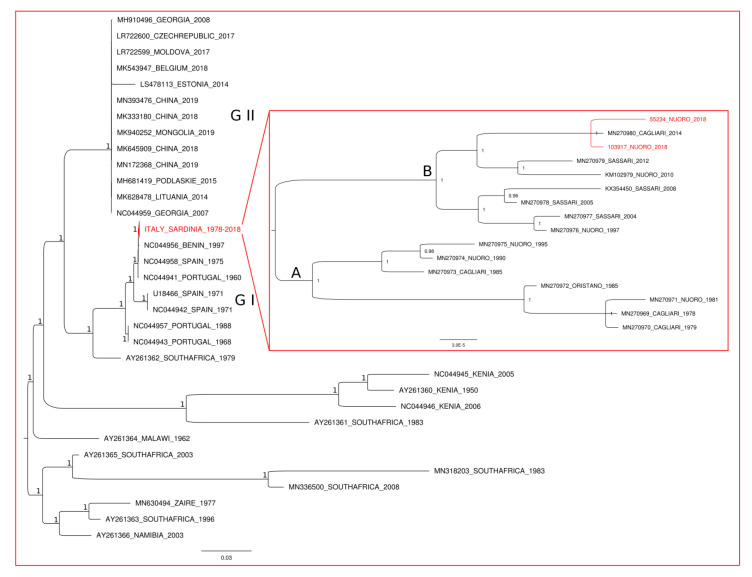
Bayesian phylogenetic tree based on ASFV complete genome sequences. The main nodes of the tree are fully supported by values of posterior probabilities = 1. The clade, including the 16 Sardinian ASFV genomes, was collapsed in the main tree and indicated with red font. In the inset are the phylogenetic relationships within the Sardinian group. The sequences isolated in the present study are indicated in red font.

Phylogenetic analysis of whole-genome sequencing data was also performed. The phylogenetic tree obtained for 48 ASFV genomes from Africa, Asia, and Europe (see the main tree in Figure 4) supports the genetic structuring already reported between the ASFV p72 genotypes I and II [6] showing two well-differentiated genetic clusters (G1 and G2 in Figure 4). The two Sardinian strains isolated in the present study were placed within the Sardinian group of sequences in the cluster G1 (which were collapsed and indicated with red font in the main phylogenetic tree of Figure 4). Among the Sardinian sequences (see the tree in the inset of Figure 4), a highly supported genetic structuring, consistent with the data of strain isolation, was observed. In particular, the Sardinian cluster splits into two main monophyletic sister groups: one (group A in Figure 4) including sequences collected from 1978 to 1995, and the second (group B in Figure 4) including the two sequences obtained in 2018 for the present study along with those from 1997 to 2014. In particular, within group B, two internal sub-groups were identified, representative of the time intervals 1997–2008 and 2010–2018, respectively. The 103917/18 and the 55234/18, from the centre of Sardinia and isolated in the present study, belong to the same sub-group of isolates 26544/OG10 (KM102979) and 97/Ot/12 (MN270979); they grouped together with a sequence (22653/Ca/2014, MN270980) from the south of the island (Cagliari) in 2014, which was the most recent among the Sardinian fully sequenced strains isolated before this study.

As seen in Table 3, the two strains isolated in 2018 from illegal free-ranging pigs were almost identical in size, number of open reading frame (ORF), and % GC, and they presented high similarity to all Sardinian isolates, without large deletions at either the left or right ends of the genome.

## 4. Discussion

The first aim of this study was to determine ASFV prevalence and distribution among free-ranging pigs in Sardinia. In the framework of the ASFV eradication program (PE-AS15-18 and subsequent additions), several depopulation actions against illegal pigs were issued in the island since December 2017, and more than 4000 illegal free-ranging pigs were culled. These pigs might have acted as a reservoir for ASFV in Sardinia, serving as a link between domestic pigs and wild boar [8]. Their interaction rate with wild boar was demonstrated to be extremely high, and recent studies reported a strong correlation between free-ranging pig numbers and ASFV outbreaks in domestic pig farms in the same municipalities [14,45,48]. Antibody prevalence and virus prevalence in these pigs were extremely high in the first year of the culling actions (2017) (61.9% and 3.2%, respectively), with the disease prevalence gradually decreasing over the years, until 1.4% and 0% of antibody-prevalence and virus-prevalence, respectively, registered in 2020. The highest antibody-prevalence was detected in the municipality with the highest free-ranging pig density (Orgosolo), while the highest virus prevalence was detected in Desulo. On the other hand, the maximum levels of the basic reproduction number (*R*_0_ ≈ 2.2 and 2.6) associated with Orgosolo and Desulo suggested the presence of a possible endemic disease context. In general, the peaks of antibody prevalence were detected in the areas where the number of illegal free-ranging pigs was higher than 300. The only exception was in Arzana, where the animals probably lived in a small area with a high contact rate, as found by Bosch et al. [45]. These results demonstrate a likelihood correlation between free-ranging pig number and both antibody and virus prevalence. This assumption is reinforced by the fact that, during subsequent culling actions, with a consequent reduction in the free-ranging pig density, the number of contacts between the three suid populations (free-ranging pigs, wild boar, and domestic pigs) decreased, and ASFV circulation dropped to zero over the years [49].

These illegal animals were apparently healthy and displayed no clinical sign of ASF at the time of culling (Dr. Sergio Masala and veterinarians of task force GIV, personal communication), despite the presence of ASFV antibodies and even ASFV genome. These “survivors” might have acted as ASFV carriers. Previous studies suggested that pigs surviving infection with moderately virulent ASFV isolates carried the virus for weeks after infection and were able to transmit the disease to naïve in contact pigs at 42 days pi [50].

The second aim of this study was to characterize the ASFV strains circulating among Sardinian illegal pigs. Due to the high presence of apparently healthy antibody-positive pigs, we hypothesized the presence of low/moderately virulent ASFV strains in the island. In the past, attenuated genotype I ASFV strains were described in Portugal (NH/P68 and OURT 88/3) and were able to confer resistance to challenge with homologous virulent isolates. These non-HAD strains were implicated in the long-term persistence of ASF in Portugal and in the presence of antibody-positive pigs in Alentejo (Portugal), without notification of clinical disease [51,52,53]. In addition, two ASFV genotype II variants with reduced virulence were recently identified in Latvia and Estonia [22,23]. We assessed the presence of non-HAD ASFV strains among free-ranging pigs in Sardinia. The immunofluorescence staining performed on all PCR^+^ Malmquist^−^ samples produced negative results, strongly suggesting the absence of non-HAD ASFV isolates in the island. Two HAD ASFV strains were instead isolates in two distinct free-ranging pigs culling activities: 55234/18 and 103917/18 (both in Nuoro province).

We initially performed in vitro studies to assess their interaction with macrophages. Macrophages are the main target population of ASFV [54], and virulent isolates developed several mechanisms to escape host immune defences in order to efficiently replicate in these cells [55]. Both “free-ranging pig” isolates presented the same growth kinetic and impact on moMФ viability as the virulent Sardinian isolate 26544/OG10, suggesting that they similarly prevent moMФ death early after infection. The 55234/18, the 103917/18, and the 26544/OG10 also had the same impact on MHC I expression as two other virulent Sardinian isolates tested in the past (22653/14 and Nu81.2). On the contrary, the attenuated NH/P68 isolate down-regulated MHC I expression on moMФ [27], which might promote NK activation in vivo [56], as we previously speculated [57]. Our data suggest that all the tested Sardinian strains, including 55234/18 and 103917/18, elude NK recognition by similar mechanisms, and overall in vitro studies did not provide any evidence of attenuation of the two isolates under investigation.

Whole-genome sequencing was then performed in order to further characterize these two ASFV isolates circulating among apparently healthy antibody-positive pigs. Before this study, 14 Sardinian isolates were fully sequenced [10,46,47], and remarkable genetic homogeneity was seen among strains collected between 1978 and 2014 [10]. Thirteen out of the 14 ASFV strains analysed by Torresi et al. (2020) [10] were collected from pigs with acute clinical signs of ASFV, whereas only one (72407/SS/05) was isolated from a hunted/found dead wild boar with unknown health status. Typing analyses of the B646L gene showed that both 103917/18 and 55234/18 belong to genotype I, in addition to all the other Sardinian ASFV isolates collected since 1978 (ASF Virus Archive, Virology, IZS of Sardinia, Sassari, Italy). Both 103917/18 and 55234/18 belong to subgroup X of the *B602L* gene and present a six amino acid repeat deletion at the C-terminal of the CD2v protein, alongside all the Sardinian isolated collected since 1990 (“modern Sardinian isolates”) [9,58]. Our phylogenetic outputs evidenced the occurrence of a high similarity between the two Sardinian strains isolated in the present study (103917/18 and 55234/18) and the other modern Sardinian ASFV whole genomes. Such a finding supports the hypothesis of a recent origin for strains 103917/18 and 55234/18, with no direct recent connection with viral strains outside the island. Our analysis revealed that the sample 22653/14, which was isolated in the southern part of the island in 2014, presented the highest similarity with both “free-ranging pig” strains. The 22653/14 is the most recent among the Sardinian fully sequenced strains isolated before this study, thus suggesting that the genomes of 103917/18 and 55234/18 might be the products of a slow and gradual evolution of Sardinian ASFV strains occurred since 1978, as described in Torresi et al. (2020) [10]. It can be hypothesized that this gradual evolution led to an attenuation of the virulence; nevertheless, recent in vivo experiments showed that infection of domestic pigs with “modern” Sardinian ASFV isolates (47/SS/08 and 26544/OG10) caused a rapid onset of ASFV clinical signs and death in domestic pigs (De Mia et al., unpublished results). Notably, intramuscular inoculation with only 10 TCID_50_ of 26544/OG10 lead to death of domestic pigs in 10–14 days, demonstrating the high virulence of the “modern” Sardinian strains (De Mia et al., unpublished results). The 103917/18 and the 55234/18 presented a similar length to all the other Sardinian strains, without deletions in either the central region or the left/right ends of the genome comparable to what was observed in Estonian ASFV strains. In fact, an attenuated ASFV variant circulating in Estonia was characterized by a deletion of 14,560 base pairs at the 5′ end and genome reorganization by duplication [23] not observed in the “free-ranging pig” strains. Our results strongly suggest the absence of ASFV immune-escape mutants circulating among free-ranging pigs in Sardinia.

Although we cannot completely rule out the presence of attenuated ASFV strains in the island, it is likely that other factors contributed to the higher resistance of free-ranging pigs to ASFV. This virus can infect feral suids, such as warthogs and bushpigs, without the development of any clinical signs [5], and it can be speculated that the higher tolerance of these free-ranging pigs has a genetic basis. A similar situation was described in western Kenya, which is an endemic ASF area. Researchers observed that a proportion of indigenous pigs were ASFV^+^ but displayed no clinical signs of infection, thus, two indigenous Kenyan pig populations were genotyped and compared to bushpigs, warthogs, European wild boars, and commercial domestic pigs. The Homa Bay pig population, where higher percentages of ASFV PCR^+^ pigs were detected, was distinct and presented a “local indigenous composition” distinct from international breeds [59]. Genetic studies are on-going in Sardinia, and more investigations are needed to define putative genetic factors behind the resilience to ASFV infection. Nevertheless, other factors might underlie the higher tolerance of these animals. These “survivor” pigs probably developed a protective immune response against the disease, and this protection could have been transmitted to the offspring through colostrum/milk. Protection to ASFV relies on multiple concomitant immune mechanisms, which are, unfortunately, largely unknown [60], but antibodies might have contributed to this protection and to the delay of ASFV dissemination in the host [61]. A study suggested that colostrum/milk from sows that survived ASFV infection had a protective effect on their offspring: seven-week-old litters (which received colostrum and milk from recovered sows) presented reduced viremia and clinical signs in response to ASFV challenge [62]. More studies are needed to better understand the role of maternal antibodies or other colostrum elements in protection to ASFV and whether this contributed to the higher ASFV-tolerance of free-ranging pigs in Sardinia. A better understanding of the immune/genetic factors underlying this ASFV tolerance might contribute to the development of effective countermeasures against ASF.

## 5. Conclusions

More than 4000 illegal free-ranging pigs were culled between 2017 and 2020 in the framework of the last ASF eradication program in Sardinia and a strong correlation between their density and antibody and virus prevalence was observed. Culling actions drastically reduce ASFV circulation among these animals. ASFV genome was identified in 53 apparently healthy antibody-positive pigs, and two HAD genotype I subtype X ASFV strains were isolated in two distinct culling actions: 55234/18 (strong PCR result, low antibody level) and 103917/18 (low PCR result, high antibody level). The two isolates were highly similar (with minor changes in the viral genome sequence) to the modern Sardinian strains, especially 22653/14. Data from this study strongly suggest the absence of non-HAD or other attenuated ASFV variants circulating among Sardinian free-ranging pigs, thus indicating that other factors contributed to the ASFV tolerance of these illegal pigs on the island.

## Figures and Tables

**Table 1:** Information on number of culled pigs, African swine fever virus (ASFV) antibody and virus prevalence in free-ranging pigs tested in Sardinia from December 2017 to February 2020.

Year	Culled Pigs	Animals Tested for Antibody Presence	Antibody-Positive Animals (Prevalence, %, CI 95%)	Animals Tested for Virus Presence	PCR-Positive Animals (Prevalence, %, CI 95%)
**2017**	616	357	61.9 (56.6–66.9)	368	3.8 (2.1–6.3)
**2018**	2652	1480	40.4 (37.9–42.9)	1703	2.2 (1.6–3.0)
**2019**	913	480	14.8 (11.7–18.3)	480	0.2 (0.0–1.1)
**2020**	303	174	14.4 (9.5–20.5)	175	0 (0–0)
**Total**	4484	2491	36.7 (34.8–38.7)	2726	1.9 (1.4–2.5)

**Table 2:** Location, number of culled and tested free-ranging pigs, ASFV antibody and virus prevalence, and estimated basic reproduction number with the associated standard error (SE).

Municipality	Culled Pigs	Animals Tested for Antibody Presence	Antibody-Positive Animals (Prevalence, %, CI 95%)	Animals Tested for Virus Presence	PCR-Positive Animals (Prevalence, %, CI 95%)	*R*_0_	SE(*R*_0_)
Orgosolo	1381	763	61.5 (57.9–64.9)	888	1.2 (0.6–2.2)	2.232	0.946
Urzulei	715	405	26.7 (22.4–31.2)	530	0 (0–0)	1.224	0.489
Villagrande Strisaili	467	262	22.1 (17.2–27.6)	269	2.2 (0.8–4.8)	1.202	0.543
Talana	388	211	43.6 (36.8–50.6)	212	5.7 (2.9–9.7)	1.339	0.599
Desulo	373	222	49.5 (42.8–56.3)	186	10.8 (6.7–16.1)	2.674	0.979
Baunei	360	206	5.3 (2.7–9.3)	204	0 (0–0)	0.422	0.182
Irgoli	304	134	0 (0–0)	134	0 (0–0)	–	–
Nuoro	199	173	0 (0–0)	199	0 (0–0)	–	–
Arzana	155	144	40.3 (32.2–48.8)	144	2.8 (0.7–6.9)	1.315	0.762

**Table 3:** Genomic sequence of the two ASFV strains isolated from free-ranging pigs (red font) and comparison with 14 fully sequenced Sardinian strains.

Strain	Size	Left ^#^	Central	Right ^#^	GC%	ORF *	Reference	GenBank Accession Number
56/Ca/78	183,636	41,212	130,415	12,009	38.56%	235	[10]	MN270969
57/Ca/79	183,639	41,214	130,415	12,010	38.56%	235	[10]	MN270970
139/Nu/81	183,645	41,220	130,415	12,010	38.56%	235	[10]	MN270971
140/Or/85	183,723	41,256	130,415	12,052	38.55%	235	[10]	MN270972
85/Ca/85	181,816	40,310	130,259	11,247	38.58%	231 ^b^	[10]	MN270973
141/Nu/90	183,720	41,333	130,241	12,146	38.52%	235	[10]	MN270974
142/Nu95	183,724	41,336	130,241	12,147	38.52%	235	[10]	MN270975
60/Nu/97	181,651	40,295	130,240	11,116	38.58%	231 ^b^	[10]	MN270976
26/Ss/04	181,869	41,762	130,240	9,867	38.58%	235	[10]	MN270977
72407/Ss/05	181,699	40,303	130,241	11,155	38.57%	231 ^b^	[10]	MN270978
47/Ss/08	184,638	41,791	130,241	12,606	38.49%	235	[46]	KX354450
26544/OG10	182,906	40,527	130,242	12,138	38.56%	235	[47]	KM102979
97/Ot/12	184,206	41,574	130,241	12,391	38.51%	235	[10]	MN270979
22653/Ca/2014	181,869	40,449	130,252	11,168	38.57%	231 ^b^	[10]	MN270980
103917/18	181,759	40,353	130,241	11,165	38.56%	231 ^b^	This study	MT932578
55234/18	181,761	40,353	130,241	11,167	38.56%	231 ^b^	This study	MT932579

^#^ Left and right refer to the left and right variable regions. The left variable region includes nucleotides from 5′-end to the beginning of A224L gene. The right variable region includes nucleotides from the end of DP238L gene to 3′ –end. Size, left, and central right are expressed in bp. * ORF, open reading frame. ^b^ KP86R, KP96L, DP93R and DP86L, located into the ITRs, are missing.

## Supplementary Materials

The following are available online at https://www.mdpi.com/2076-393X/8/3/549/s1, Figure S1: Images of apparently healthy illegal pigs, Figure S2: Threshold cycle values of real-time PCR+ free-ranging pig samples. Figure S3: ASFV antibodies of Malmquist^+^ free-ranging pigs, Figure S4: Haemoadsorbing effect of 103917/18 and 55234/18. Figure S5: Amino acid sequence of partial *B602L* gene of Sardinian strains. Figure S6: Amino acid sequence of partial *EP402R* gene of Sardinian strains. Table S1: Geographic origin, sample source, and collection data of samples used in this study. Table S2: Information on ASF virus+ free-ranging pigs, including municipality and month of the culling action, virological (tested organs, Ct values of real-time PCR, Malmquist) and serological (ELISA and immunoblotting) results.

## References

[1] Sanchez-Cordon P.J., Montoya M., Reis A.L., Dixon L.K. (2018). African swine fever: A re-emerging viral disease threatening the global pig industry. Vet. J..

[2] Iglesias I., Martínez M., Montes F., de la Torre A. (2019). Velocity of ASF spread in wild boar in the European Union (2014–2017). Int. J. Infect. Dis..

[3] OIE, WAHIS Interface. https://www.oie.int/wahis_2/public/wahid.php/Wahidhome/Home.

[4] Dixon L.K., Sun H., Roberts H. (2019). African Swine Fever. Antiviral Res..

[5] Netherton C., Connell S., Benfield C.T.O., Dixon L.K. (2019). The genetic of life and death: Virus-host interactions underpinning resistance to African swine fever, a viral hemorrhagic disease. Front. Genet..

[6] Bastos A.D., Penrith M.L., Cruciere C., Edrich J.L., Hutchings G., Roger F., Couacy-Hymann E.R., Thomson G. (2003). Genotyping field strains of African swine fever virus by partial p72 gene characterisation. Arch. Virol..

[7] Costard S., Wieland B., de Glanville W., Jori F., Rowlands R., Vosloo W., Roger F., Pfeiffer D.U., Dixon L.K. (2009). African swine fever: How can global spread be prevented?. Philos. Trans. R. Soc. B.

[8] Loi F., Cappai S., Coccolone A., Rolesu S. (2019). Standardized risk analysis approach aimed to evaluate the last standardized risk analysis approach aimed to evaluate the last African swine fever eradication program performance in Sardinia. Front. Vet. Sci..

[9] Sanna G., Dei Giudici S., Bacciu D., Angioi P.P., Giammarioli M., De Mia G.M., Oggiano A. (2017). Improved strategy for molecular characterization of African swine fever virus from Sardinia, based on analysis of p30, CD2V and I73R/I329L variable regions. Transbound. Emerg. Dis..

[10] Torresi C., Fiori M., Bertolotti L., Floris M., Colitti B., Giammarioli M., Dei Giudici S., Oggiano A., Malmberg M., De Mia G. (2020). The evolution of African swine fever virus in Sardinia (1978–2014) as revealed by whole-genome sequencing and comparative analysis. Transbound. Emerg. Dis..

[11] Fois F., Culurgioni J., Cappai S., Piras P.M., Toma L., Rolesu S., Liciardi M. (2016). An overview on Sardinia’s soft ticks (Ixodida: Argasida). Exp. Appl. Acarol..

[12] Sánchez R., Oleaga A., Sánchez-Vizcaíno J.M. (2016). Serological surveillance and direct field searching reaffirm the absence of ornithodoros erraticus ticks role in african swine fever cycle in Sardinia. Transbound. Emerg. Dis..

[13] Mur L., Atzeni M., Martínez-López B., Feliziani F., Rolesu S., Sanchez-Vizcaino J.M. (2016). Thirty-Five-Year Presence of African Swine Fever in Sardinia: History, Evolution and Risk Factors for Disease Maintenance. Transbound. Emerg. Dis..

[14] Cappai S., Rolesu S., Coccollone A., Laddomada A., Loi F. (2018). Evaluation of biological and socio-economic factors related to persistence of African swine fever in Sardinia. Prev. Vet. Med..

[15] Jurado C., Fernandez-Carrion E., Mur L., Rolesu S., Laddomada A., Sanchez-Vizcaino J.M. (2018). Why is African swine fever still present in Sardinia?. Transbound. Emerg. Dis..

[16] Laddomada A., Rolesu S., Loi F., Cappai S., Oggiano A., Madrau M.P., Sanna M.L., Pilo G., Bandino E., Brundu D. (2019). Surveillance and control of African Swine Fever in free-ranging pigs in Sardinia. Transbound. Emerg. Dis..

[17] Penrith M.L., Thomson G.R., Bastos A.D.S., Phiri O.C., Lubisi B.A., Du Plessis E.C., Macome F., Pinto F., Botha B., Esterthuysen J. (2004). An investigation into natural resistance to African swine fever in domestic pigs from an endemic area in southern Africa. Rev. Sci. Tech. Off. Int. Epiz..

[18] Atuhaire D.K., Afayoa M., Ochwo S., Mwesigwa S., Mwiine F.N., Okuni J.B., Olaho-Mukani W., Ojok L. (2013). Prevalence of African swine fever in apparently healthy domestic pigs in Uganda. BMC Vet. Res..

[19] Patrick B.N., Machuka E.M., Githae D., Banswe G., Amimo J.O., Ongus J.R., Masembe C., Bishop R.P., Steinaa L., Djikeng A. (2020). Evidence for the presence of African swine fever virus in apparently healthy pigs in South-Kivu province of Democratic Republic of Congo. Vet. Microbiol..

[20] Wozniakowski G., Kozak E., Kowalczyk A., Lyjak M., Pomorska-Mol M., Niemczuk K., Pejsak Z. (2016). Current status of African swine fever virus in a population of wild boar in eastern Poland (2014–2015). Arch. Virol..

[21] Nurmoja I., Petrov A., Breidenstein C., Zani L., Forth J.H., Beer M., Kristian M., Viltrop A., Blome S. (2017). Biological characterization of African swine fever virus genotype II strains from north-eastern Estonia in European wild boar. Transbound. Emerg. Dis..

[22] Gallardo C., Soler A., Rodze I., Nieto R., Cano-Gomez C., Fernarndo-Pinero J., Arias M. (2019). Attenuated and non-haemoadsorbing (non-HAD) genotype II African swine fever virus (ASFV) isolated in Europe, Latvia 2017. Transbound. Emerg. Dis..

[23] Zani L., Forth J.H., Forth L., Nurmoja I., Leidenberger S., Henke J., Carlson J., Breidenstein C., Viltrop A., Hoper D. (2018). Deletion at the 5′-end of Estonian ASFV strains associated with an attenuated phenotype. Sci. Rep..

[24] World Organization for Animal Health (OIE) (2019). Manual of Diagnostic Tests and Vaccines for Terrestrial Animals 2019. Chapter 3.8.1—African Swine Fever (Infection with African Swine Fever Virus).

[25] King D.P., Reis S.M., Hutchings G.H., Grieson S.S., Wilkinson P.J., Dixon L.K., Bastos A.D.S., Drew T.W. (2003). Development of a TaqMan PCR assay with internal amplification control for the detection of African swine fever virus. J. Virol. Methods.

[26] Franzoni G., Graham S.P., Sanna G., Angioi P., Fiori M.S., Anfossi A., Amadori M., Dei Giudici S., Oggiano A. (2018). Interaction of porcine monocyte-derived dendritic cells with African swine fever viruses of diverse virulence. Vet. Microbio..

[27] Franzoni G., Razzuoli E., Dei Giudici S., Carta T., Galleri G., Zinellu S., Ledda M., Angioi P., Modesto P., Graham S.P. (2020). Comparison of macrophage responses to African swine fever viruses reveals that the NH/P68 strain is associated with enhanced sensitivity to type I IFN and cytokine responses from classically associated macrophages. Pathogens.

[28] Illumina bcl2fastq Tool. https://support.illumina.com/sequencing/sequencing_software/bcl2fastq-conversion-software.html?langsel=/us/.

[29] TrimGalore Tool. https://github.com/FelixKrueger/TrimGalore.

[30] Groenen M.A., Archibald A.L., Uenishi H., Tuggle C.K., Takeuchi Y., Rothschild M.F., Claire R.G., Chankyu P., Denis. M., Hendrik. J.M. (2012). Analyses of pig genomes provide insight into porcine demography and evolution. Nature.

[31] Li H., Durbin R. (2009). Fast and accurate short read alignment with Burrows-Wheeler transform. Bioinformatics.

[32] Li H. (2011). A statistical framework for SNP calling, mutation discovery, association mapping and population genetical parameter estimation from sequencing data. Bioinformatics.

[33] Picard Toolkit. https://broadinstitute.github.io/picard/.

[34] Freebayes GitHub Repository. https://arxiv.org/abs/1207.3907.

[35] Katoh K., Standley D.M. (2013). MAFFT multiple sequence alignment software Version 7: Improvements in performance and usability. Mol. Biol. Evol..

[36] Okonechnikov K., Golosova O., Fursov M., the UGENE team (2012). Unipro UGENE: A unified bioinformatics toolkit. Bioinformatics.

[37] Ronquist F., Teslenko M., van der Mark P., Ayres D.L., Darling A., Höhna S., Larget B., Liu L., Suchard M.A., Huelsenbeck J.P. (2012). MrBayes 3.2: Efficient Bayesian phylogenetic inference and model choice across a large model space. Syst. Biol..

[38] 38. Miller M.A., Pfeiffer W., Schwartz T. Creating the CIPRES Science Gateway for inference of large phylogenetic trees. Proceedings of the Gateway Computing Environments Workshop (GCE).

[39] Gelman A., Rubin D.B. (1992). Inference from iterative simulation using multiple sequences. Stat. Sci..

[40] Scarpa F., Sanna D., Cossu P., Lai T., Casu M., Curini-Galletti M. (2019). How to achieve internal fertilization without a vagina: The study case of the genus Archilina Ax, 1959 (Platyhelminthes, Proseriata) from Canary Islands. Mar. Biodivers..

[41] FigTree 1.4.0. http://tree.bio.ed.ac.uk/software/figtree/.

[42] Kumar S., Stecher G., Tamura K. (2016). MEGA7: Molecular Evolutionary Genetics Analysis version 7.0 for bigger datasets. Mol. Biol. Evol..

[43] Bailey N.T.J. (1975). The Mathematical Theory of Infectious Diseases and Its Applications.

[44] Becker N. (1989). Analysis of Infectious Disease Data.

[45] Bosch J., Barasona J.A., Cadenas-Fernandez E., Jurado C., Pintore A., Denurra D., Cherchi M., Vicente J., Sanchez-Vizcaıno J.M. (2020). Retrospective spatial analysis for African swine fever in endemic areas to assess interactions between susceptible host populations. PLoS ONE.

[46] Granberg F., Torresi C., Oggiano A., Malmberg M., Iscaro C., De Mia G.M., Belak S. (2016). Complete genome sequence of an African swine fever virus isolate from Sardinia, Italy. Genome Annouc..

[47] Bacciu D., Deligios M., Sanna G., Madrau M.P., Sanna M.L., Dei Giudici S., Oggiano A. (2016). Genomic analysis of sardinian 26544/OG10 isolate of African swine fever virus. Virol. Rep..

[48] Cadenas-Fernandez E., Sanchez-Vizcaino J.M., Pintore A., Denurra D., Cherchi M., Jurado C., Vicente J., Barasona J.A. (2019). Free-ranging pig and wild boar interactions in an endemic area of African swine fever. Front. Vet. Sci..

[49] Laddomada A. (2020). The last mile in the eradication of ASF in Sardinia. OIE Bull..

[50] Eblè P.L., Hagenaars J., Weesendorp E., Quark S., Moonen-Leusen H.W., Loeffen W.L.A. (2019). Trasmission of African Swine Fever Virus via carrier (survivor) pigs does occour. Vet. Microbiol..

[51] Vigario J.D., Terrinha A.M., Moura Nunes J.F. (1974). Antigenic relationship among strains of African swine fever virus. Arch. Ges. Virusforsch.

[52] Boinas F.S., Hutchings G.H., Dixon L.K., Wilkinson P.J. (2004). Characterization of pathogenic and non-pathogenic African swine fever virus isolates from Ornithodoros erraticus inhabiting pig premises in Portugal. J. Gen. Virol..

[53] Arias M., de la Torre A., Dixon L., Gallardo C., Jori F., Laddomada A., Martins C., Parkhouse R.M., Revilla Y., Rodriguez F.M. (2017). Approaches and Perspectives for Development of African Swine Fever Virus Vaccines. Vaccines.

[54] Sánchez-Cordón P.J., Romero-Trevejo J.L., Pedrera M., Sánchez-Vizcaíno J.M., Bautista M.J., Gómez-Villamandos J.C. (2008). Role of hepatic macrophages during the viral haemorrhagic fever induced by African swine fever virus. Histol. Histopathol..

[55] Dixon L.K., Sánchez-Cordón P.J., Galindo I., Alonso C. (2017). Investigations of Pro- and Anti-Apoptotic Factors Affecting African Swine Fever Virus Replication and Pathogenesis. Viruses.

[56] Lanier L.L. (2005). NK cell recognition. Annu. Rev. Immunol..

[57] Franzoni G., Dei Giudici S., Oggiano A. (2018). Infection, modulation and responses of antigen-presenting cells to African swine fever viruses. Virus Res..

[58] Giammarioli M., Gallardo C., Oggiano A., Iscaro C., Nieto R., Pellegrini C., Dei Giudici S., Arias M., De Mia G.M. (2011). Genetic characterisation of African swine fever viruses from recent and historical outbreaks in Sardinia (1978–2009). Virus Genes.

[59] Mujibi F.D., Okoth E., Cheruiyot E.K., Onzere C., Bishop R.P., Fevre E.M. (2018). Genetic diversity, breed composition and admixture of Kenyan domestic pigs. PloS ONE.

[60] Carlson J., O’Donnel V., Alfano M., Salinas L.V., Holinka L.G., Krug P.W., Gladue D.P., Higgs S., Borca M.V. (2016). Association of the host immune response with protection using a live attenuated African swine fever virus model. Viruses.

[61] Escribano J.M., Galindo I., Alonso C. (2013). Antibody-mediated neutralization of African swine fever virus: Myths and facts. Virus Res..

[62] Schlafer D.H., McVicar J.W., Mebus C.A. (1984). African swine fever convalescent sows: Subsequent pregnancy and the effect of colostral antibody on challenge inoculation of their pigs. Am. J. Vet. Res..

