# Supplementary material for: African Swine Fever Circulation among Free-Ranging Pigs in Sardinia: Data from the Eradication Program
Source: Vaccines (Basel). 2020 Sep 21;8(3):549. doi: 10.3390/vaccines8030549 (PMC7563918; doi:10.3390/vaccines8030549)
Supplement: Supplementary file 1 [file vaccines-08-00549-s001.pdf]

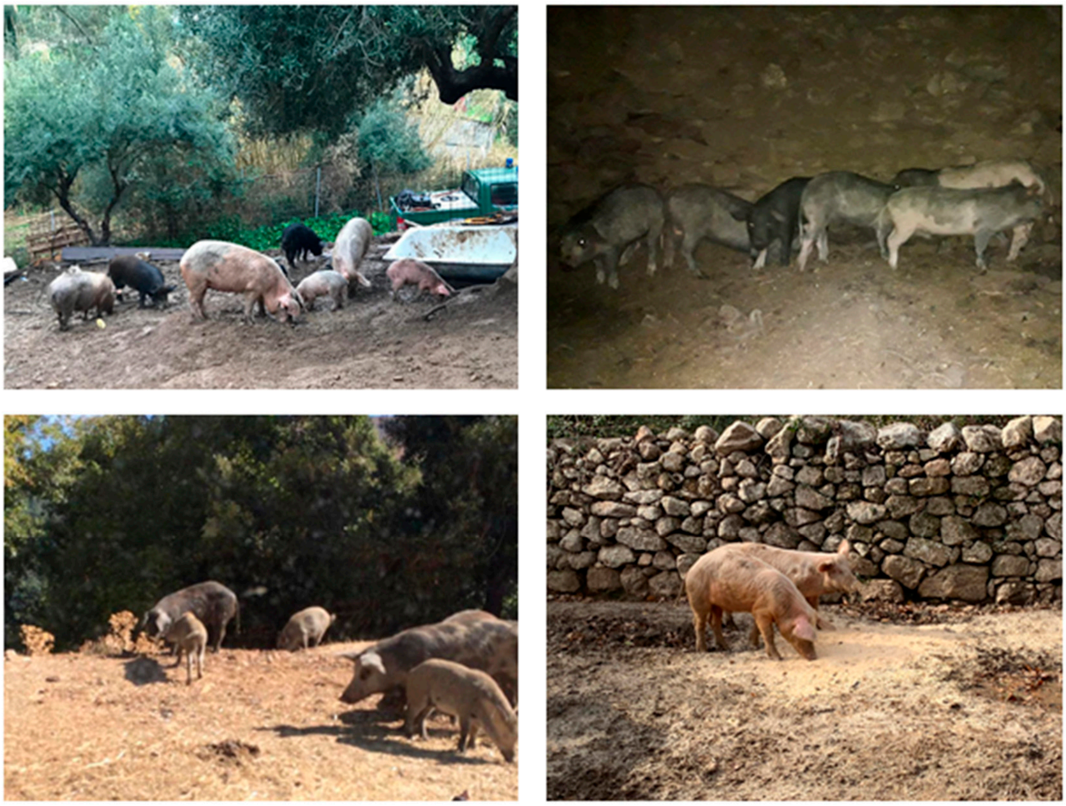

Figure S1. Images of apparently healthy free-ranging pigs (kindly provided by Dr. Sergio Masala, AHS).

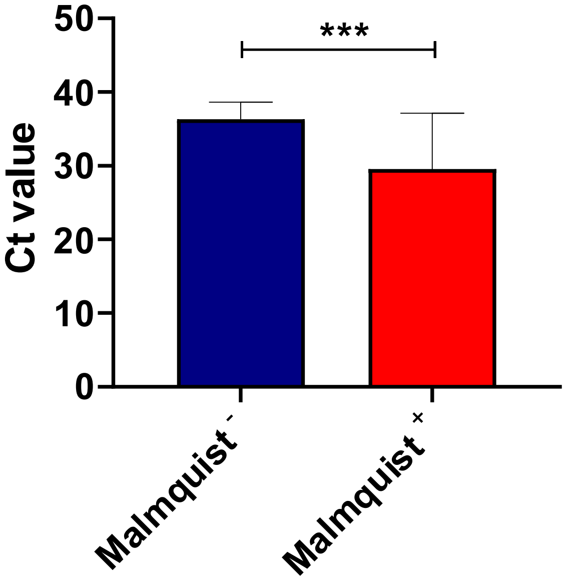

Figure S2. Threshold cycle values of real-time PCR+ free-ranging pig samples. Threshold cycle (Ct) values of Malmquist+ and Malmquist- samples were compared using a student T test; *** $p < 0.001$. Mean values and SD are shown.

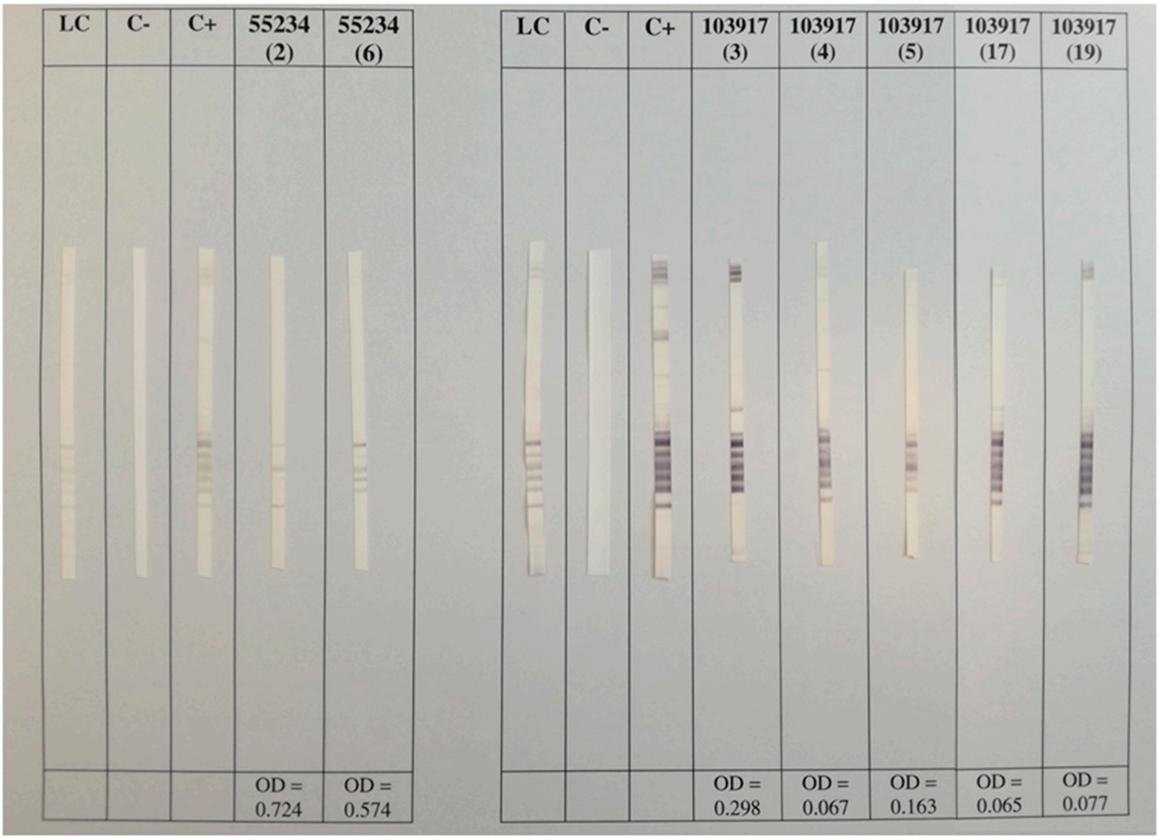

Figure S3. ASFV-antibodies of Malmquist+ free-ranging pigs. Immunoblotting strips incubated with the sera of the Malmquist+ free-ranging pigs culled in Desulo (55234) or Talana (103917), alongside positive (C+), negative (C-) and low positive (LC) controls.

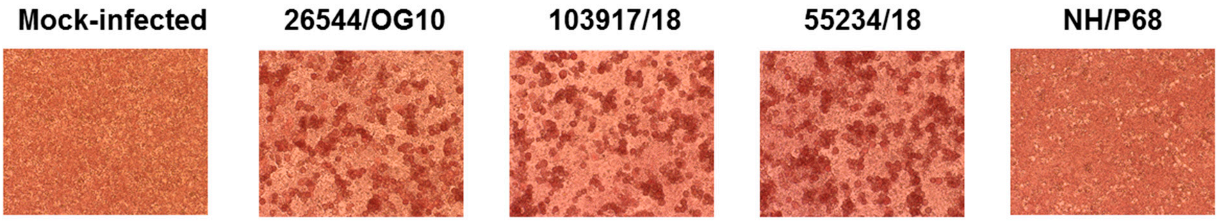

Figure S4. Haemoadsorbing effect of 103917/18 and 55234/18. Two days old monocytes were infected with the virulent HAD 26544/OG10, the two tested strains (103917/18 and 55234/18), and the non-HAD NH/P68, alongside mock-infected controls. Autologous erythrocytes were added to culture media; 24 hpi haemoadsorption effect was visible for 26544/OG10, 103917/18 and 55234/18.

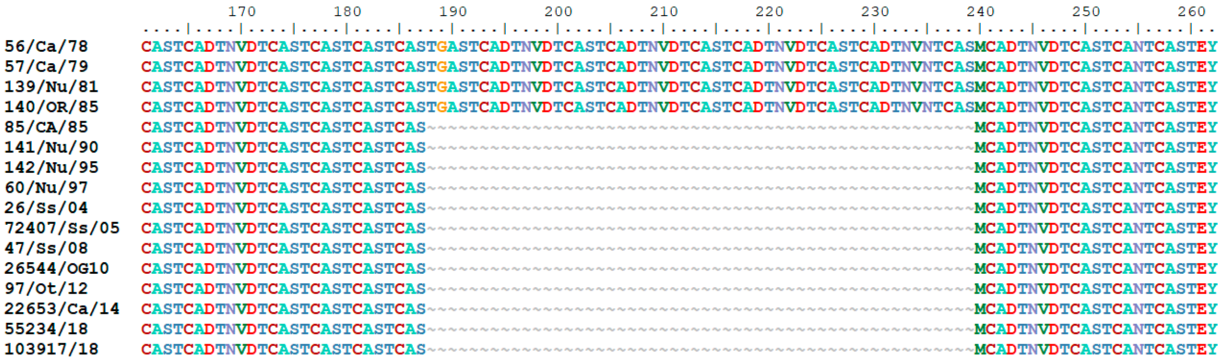

Figure S5. Amino acid sequence of partial B602L gene of Sardinian strains. Amino acid sequence of partial B602L gene of 55234/18 and 103917/18 strains, compared with other Sardinian isolates reported in Table 4.

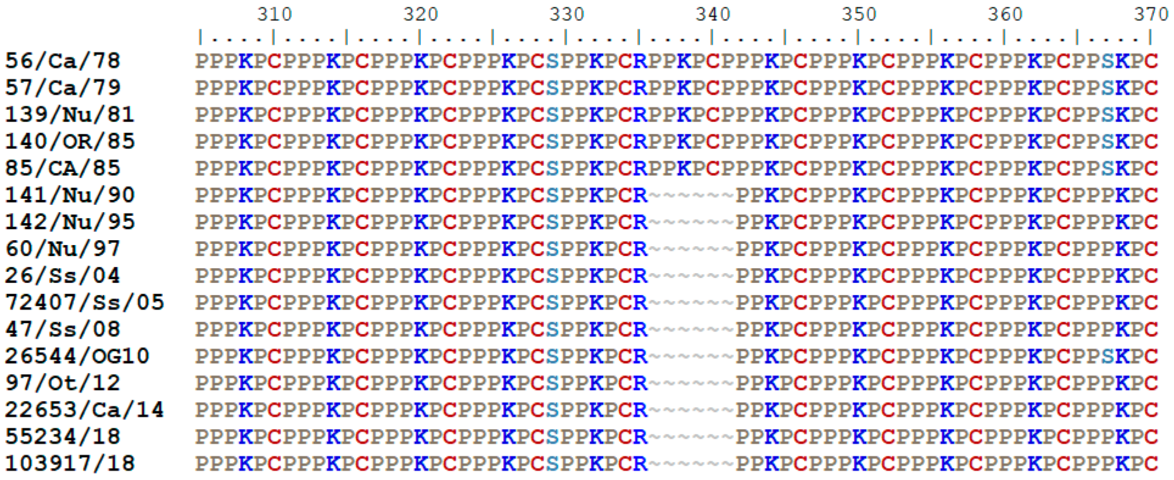

Figure S6. Amino acid sequence of partial EP402R gene of Sardinian strains. Amino acid sequences of partial EP402R gene of 55234/18 and 103917/18, compared with other Sardinian isolates reported in Table 4.

Table S1. Geographic origin, sample source and collection data of samples used in this study.

Strain	Year	Country	Host species	GenBank accession number
103917/18	2018	Sardinia (Italy)	Domestic pig (free-ranging)	MT932578
55234/18	2018	Sardinia (Italy)	Domestic pig (free-ranging)	MT932579
22653/Ca/2014	2014	Sardinia (Italy)	Domestic pig	MN270980
97/Ot/12	2012	Sardinia (Italy)	Domestic pig	MN270979
26544/OG10	2010	Sardinia (Italy)	Domestic pig	KM102979
47/Ss/08	2008	Sardinia (Italy)	Domestic pig	KX354450
72407/Ss/2005	2005	Sardinia (Italy)	Wild Boar	MN270978
26/Ss/04	2004	Sardinia (Italy)	Domestic pig	MN270977
60/Nu/97	1997	Sardinia (Italy)	Domestic pig	MN270976
142/Nu/95	1995	Sardinia (Italy)	Domestic pig	MN270975
141/Nu/90	1990	Sardinia (Italy)	Domestic pig	MN270974
85/Ca/85	1985	Sardinia (Italy)	Domestic pig	MN270973

140/Or/85	1985	Sardinia (Italy)	Domestic pig	MN270972
139/Nu/81	1981	Sardinia (Italy)	Domestic pig	MN270971
57/Ca/79	1979	Sardinia (Italy)	Domestic pig	MN270970
56/Ca/78	1978	Sardinia (Italy)	Domestic pig	MN270969
BA71	1971	Spain	Domestic pig	NC044942
BA71V		Spain	Tissue culture adapted (Vero)	U18466
Benin 97/1	1997	Republic of Benin (West Africa)	Domestic pig	NC044956
E75	1975	Spain	Domestic pig	NC044958
L60	1960	Portugal	Domestic pig	NC044941
NH/P68	1968	Portugal	Domestic pig	NC044943
OUTR 88/3	1988	Portugal	Tick	NC044957
Belgium/Etalle/wb/2018	2018	Belgium	Wild Boar	MK543947
Pig/HLJ/2018	2018	China	Domestic pig	MK333180
ASFV-wbBS01	2019?	China	Wild Boar	MK645909
ASFV/pig/China/CAS19-01/2019	2019	China	Domestic pig	MN172368
Moldova 2017/1	2017	Moldova	Wild Boar	LR722599
ASFV/POL/2015/Podlaskie	2015	Poland	Wild Boar	MH681419
ASFV CzechRepublic 2017/1	2017	Czech Republic	Wild Boar	LR722600
Estonia 2014	2014	Estonia	Wild Boar	LS478113
ASFV/LT14/1490	2014	Lithuania	Wild Boar	MK628478
ASFV Wuhan 2019-1	2019	China	Domestic pig	MN393476
Georgia 2007/1	2007	Georgia	Domestic pig	NC044959
Ken06.Bus	2006	Kenya	Domestic pig	NC044946
Ken05/Tk1	2005	Kenya	Tick	NC044945
Zaire	1977	Zaire	Domestic pig	MN630494
RSA_2_2008	2008	South Africa	Tick	MN336500
LIV_5_40	1983	Zambia	Tick	MN318203
CN/2019/InnerMongolia-AES01	2019	Mongolia	Wild Boar	MK940252
Georgia 2008/2	2008	Georgia	Domestic pig	MH910496
Warthog	1980	Namibia	Warthog	AY261366
Warmbaths	1987	Republic of South Africa	Tick	AY261365
Tengani 62	1962	Malawi	Domestic pig	AY261364
Pretorisuskop/96/4	1996	Republic of South Africa	Tick	AY261363
Mkuzi 1979	1979	Zululand	Tick	AY261362
Malawi Lil-20-1 (1983)	1983	Malawi	Tick	AY261361
Kenya 1950	1950	Kenya	Domestic pig	AY261360

Table S2. Information on ASF virus+ free-ranging pigs, including municipality and month of the culling action, virological (tested organs, Ct values of real-time PCR, Malmquist) and serological (ELISA and immunoblotting) results.

Culling action (location and month)	Identification number	Organ	Ct value*	Malmquist	ELISA	IB#

Desulo December 2017	96355 (12)	spleen	39.1	neg	pos	pos
	96355 (13)	spleen	37.68	neg	pos	pos
	96355 (40)	spleen	37.75	neg	pos	pos
	96355 (49)	spleen	35.05	neg	pos	pos
Arzana December 2017	96777 (1)	spleen	38.54	neg	pos	pos
	96777 (8)	spleen	35.85	neg	neg	/
	96777 (16)	spleen	33.39	neg	neg	/
	96777 (18)	spleen	33.37	neg	pos	pos
Orgosolo (Montes) December 2017	96804 (13)	spleen	38.82	neg	pos	pos
Villagrande Strisaili December 2017	101337 (1)	spleen	37.53	neg	pos	pos
	101337 (16)	spleen	38.59	neg	pos	pos
	101337 (17)	spleen	37.63	neg	pos	pos
	101337 (27)	spleen	38.04	neg	neg	/
Orgosolo (Pradu) December 2017	103306 (39)	spleen	37.7	neg	neg	/
Orgosolo (Montes) January 2018	2428 (48)	spleen	37.8	neg	neg	/
	2428 (81)	spleen	37.63	neg	pos	pos
	2428 (84)	spleen	37.4	neg	pos	pos
	2428 (102)	spleen	35.49	neg	pos	pos
Orgosolo (Montes) January 2018	5703 (103)	spleen	38.9	neg	pos	pos
	5703 (105)	spleen	38.3	neg	neg	/
Orgosolo (Biriddi - Iserrai) February 2018	18285 (19)	spleen	38.11	neg	pos	pos
	19185 (1)	lymph node	33.01	neg	pos	pos
	19185 (2)	lymph node	37.4	neg	pos	pos
Desulo June 2018	55232 (2)	spleen	37.33	neg	pos	pos
	55232 (6)	spleen	32.68	neg	pos	pos
	55232 (9)	spleen	34.86	neg	pos	pos
	55232 (10)	spleen	37.85	neg	pos	pos
Desulo June 2018	55234 (1)	spleen	20.44	pos	neg	/
	55234 (2)	spleen	22.99	pos	pos	pos
	55234 (3)	spleen	32.01	neg	pos	pos
	55234 (4)	spleen	34.86	neg	pos	pos
	55234 (5)	spleen	19.1	pos	neg	/
	55234 (6)	spleen	23.85	pos	pos	pos
	55234 (7)	spleen	31.64	neg	neg	/
	55234 (8)	spleen	31.78	neg	pos	pos
	55234 (9)	spleen	31.7	neg	neg	/
	55234 (10)	spleen	37.37	neg	pos	pos
	55234 (11)	spleen	35.58	neg	pos	neg
Talana November 2018	90280 (10)	spleen	38.1	neg	pos	pos
	90280 (18)	spleen	34.79	neg	pos	pos
	90350 (40)	spleen	36.72	neg	pos	pos
Villagrande (Luli) November 2018	90307 (4)	spleen	35.75	neg	pos	pos
Villagrande (Carcaredda) November 2018	90335 (10)	spleen	37.93	neg	pos	pos
Talana December 2018	103917 (3)	spleen	27.14	pos	pos	pos
	103917 (4)	spleen	34.84	pos	pos	pos
	103917 (5)	spleen	36.67	pos	pos	pos

	103917 (8)	lung	33.14	neg	pos	pos
	103917 (14)	lung	37.98	neg	pos	pos
	103917 (16)	lung	35.8	pos	neg	/
	103917 (17)	spleen	36.95	pos	pos	pos
	103917 (19)	spleen	37.98	pos	pos	pos
	103917 (20)	spleen	39.3	neg	pos	pos
Desulo January 2019	13750 (1)	spleen	37.79	neg	pos	pos

*Ct: threshold cycle; #IB: immunoblotting; pos: positive; neg: negative; '/': not performed (IB was performed only on ELISA positive or inconclusive samples).
